# It’s Got Too Greedy. New Therapeutic Options for Metabolic[ally] Addicted NSCLC?

**DOI:** 10.3390/cancers12113223

**Published:** 2020-11-01

**Authors:** Elisa Caiola, Massimo Broggini

**Affiliations:** Laboratory of Molecular Pharmacology, Istituto di Ricerche Farmacologiche Mario Negri IRCCS, 20156 Milan, Italy; elisa.caiola@marionegri.it

Accounting for more than 2 million new cases and around 1.8 million deaths worldwide, lung cancer is the leading cause of cancer-related death [1]. Cigarette smoke is the main risk factor involved in the etiology of non-small-cell lung cancer (NSCLC), the most frequent histologic subtype. According to current estimates, more than 80% of cases are a consequence of this habit [2].

Besides all the strategies to prevent tobacco use to reduce lung cancer incidence, clinical practice has been increasingly moving towards personalized management of patients affected by this malignancy, to improve the outcomes. 

A very high mutational load characterizes NSCLC, giving rise to great tumor heterogeneity [3] and the histological classification of NSCLC per se is not sufficient for the selection of the most effective therapy to patients. The molecular characterization is now required to refine NSCLC diagnosis and define specific subgroups of patients with peculiar vulnerabilities.

The identification of targetable alterations, such as in *EGFR* and *ALK*, has radically changed the management of patients affected by this subgroup of NSCLC. EGFR-, ALK- and ROS-altered tumors are in fact extremely sensitive to EGFR inhibitors (such as erlotinib, gefitinib and afatinib) and ALK/ROS inhibitors (crizotinib, ceritinib, alectinib, brigantinib), respectively. In NSCLC patients, these targeted therapies have shown higher progression-free survival and safer toxicity profiles than the standard regimen [4,5,6,7].

Furthermore, immune checkpoint inhibitors (pembrolizumab, nivolumab, atezolizumab, durvalumab), which restore the T cell-mediated antitumor immune response, and have recently shown impressive results in the outcomes with easier-to-manage adverse effects in NSCLC patients expressing ‘immune’ markers (for example PD-1 and PD-L1) [8,9,10]. 

Patients carrying these targetable alterations, however, represent only a small portion of all NSCLCs, so there is an urgent need to find out other peculiar weaknesses of the disease for the remaining majority of patients.

In the mutational spectrum of NSCLCs, LKB1-mutated tumors represent about the 30% of the cases [11]. The *LKB1* tumor suppressor gene encodes for a serine/threonine kinase acting as a master regulator of cell energy homeostasis [12,13]. The LKB1-controlled signaling pathway has an important role in restoring energy homeostasis by activating catabolic pathways and inhibiting anabolic metabolism when energetics stress occurs and intracellular ATP production decreases [14,15]. In particular, AMPK, one of the major targets of LKB1, suppresses mammalian target of rapamycin complex 1 (mTORC1) [15], a central integrator of nutrient and growth factor in human cancers. AMPK also regulates redox processes by restoring not only ATP, but also NADPH, which is used to neutralize radical oxygen species (ROS) arising during metabolic stress [16]. The pivotal role and the frequency of *LKB1* inactivating mutations in NSCLC makes LKB1 an attractive target in cancer.

At present, LKB1 status-driven treatment choice is still an unmet clinical need. First, almost invariably, *LKB1* mutations result in the absence of the protein [17] and hence in the lack of a direct cancer-associated target. Second, LKB1 mutations are mutually exclusive with targetable alterations so that LKB1ness patients cannot receive TKIs targeted therapies [18]. Third, LKB1 null status has emerged as a major determinant of the ‘cold’ immune microenvironment in NSCLC, determining poor response to immune checkpoint inhibitors [17]. Conversely, *LKB1* alterations are common events in KRAS mutant tumors [19]. After several efforts to target mutant KRAS, G12C KRAS inhibitors are undergoing early-phase clinical trials with impressive results [20,21] and will represent in the near future a turning point to treat specific KRAS mutant tumors. Nevertheless, there is cumulating evidence that in the defined subgroup of oncogenic KRAS NSCLC, ‘intradriver heterogeneity’ also exists [18] and the co-occurring alterations (as in *LKB1* gene) could be very impactful on this aspect. In addition, other (than G12C) KRAS substitutions remain undruggable, so alternative strategies to treat these subpopulations are required.

Discovered more than 100 years ago, metabolic rewiring is more and more recognized as one of the hallmarks of cancer. Defects in cancer cell metabolism can be helpful for the diagnosis or monitoring of growth, but also for possible new treatments. The latter is of particular interest, with the notion that specific metabolic dependencies can represent a vulnerable point for different cancer types. This is witnessed by the appearance in clinical trials of inhibitors of specific metabolic pathways altered in cancer.

In this context, altered KRAS and LKB1 have been described as key factors in promoting metabolic reprogramming. In NSCLC, oncogenic *KRAS* promotes oxidative phosphorylation through glucose metabolism stimulation and glutamine metabolism enhancement [22,23]. LKB1 loss, instead, drives hypoxia inducible factor (HIF) signaling increase, thus promoting aerobic glycolysis and reducing oxidative phosphorylation dependency [24].

Furthermore, as our and other groups reported, the co-existence of the two alterations further accelerates cell metabolism compared to the single mutations, by exploiting both glycolysis and oxidative phosphorylation, thus rendering the tumor particularly sensitive to nutrient deprivation (as schematically represented in Figure 1) ([5,6,7,8,9,10,11,12,13,14,15,16,17,18,19,20,21,22,23,24,25,26,27,28]. 

Altogether, this evidence clearly indicates that rapidly growing tumors, with high metastatic potential and with low response to treatment (as KRAS/LKB1 mutated NSCLCs are), are also highly dependent on nutrient availability. This offers new encouragement in this area, with the possibility to test nutrient deprivation (through low calories diets) [29] and/or metabolic inhibitors (glutaminase inhibitors, metformin, fatty acid synthesis inhibitors) in combination with chemotherapy and immunotherapy to increase the chance of survival of these patients with very low prognosis. In vitro data are particularly encouraging and new efforts should be directed in defining additional vulnerable points in the metabolic cascades in NSCLC (as well as in other cancer types) to design new, safe, and efficacious inhibitors for a rapid inclusion in future clinical trials. 

## Figures and Tables

**Figure 1:**
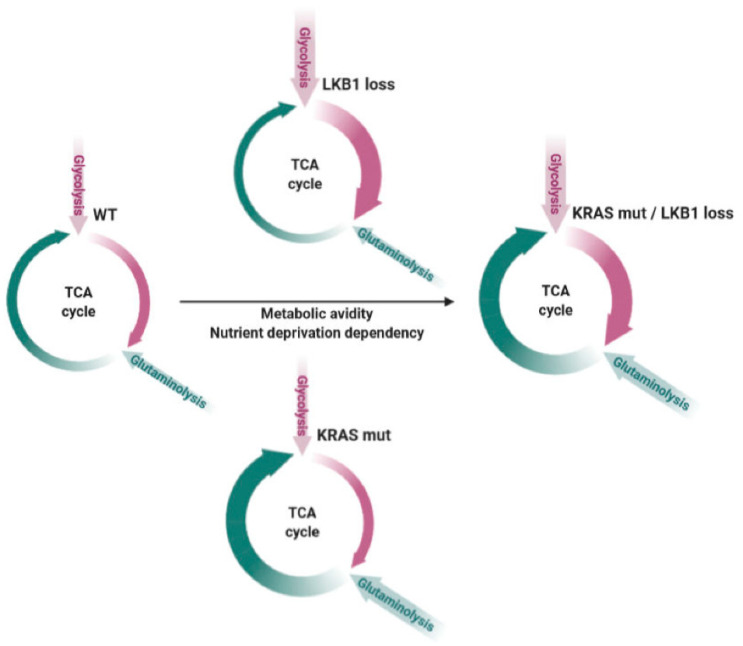
Schematic overview summarizing the enhanced metabolic avidity and nutrient deprivation sensitivity of KRAS and LKB1 co-mutated tumors compared to wild type (WT) or single mutated NSCLCs. Violet arrows refer to glycolysis and its exploitation in the tricarboxylic acid (TCA) cycle, while green arrows represent glutaminolysis and the TCA cycle fueled by this process. The thickness of the arrows shows the dependency of the two different processes according to the different backgrounds. The black arrow represents the increase in metabolic avidity and nutrient deprivation dependency according to the different genotypes. Adapted from [25].
